# Trauma or growth after a natural disaster? The mediating role of rumination processes

**DOI:** 10.3402/ejpt.v6.26557

**Published:** 2015-07-31

**Authors:** Felipe E. García, Félix Cova, Paulina Rincón, Carmelo Vázquez

**Affiliations:** 1 Facultad de Ciencias Sociales, Universidad Santo Tomás, Concepción, Chile; 2 Psychology Department, University of Concepcion, Concepción, Chile; 3 School of Psychology, Complutense University, Madrid, Spain

**Keywords:** Benefit-finding, distress, earthquake, natural disaster, rumination, posttraumatic stress symptoms, tsunami

## Abstract

The aim of this study was to test a cognitive model of posttraumatic symptoms (PTS) and posttraumatic growth (PTG) after exposure to a natural disaster. It was hypothesized that although subjective severity of trauma would be related to the severity of PTS, this relation would be mediated by brooding and cognitive strategies related to the presence of repetitive negative content in thoughts. Furthermore, the relation between severity and PTG would be fully mediated by deliberate rumination (DR), cognitive strategies related to conscious efforts focused on handling the event. To evaluate the cognitive model, adults (*N*=351) who lost their homes as a result of the earthquake and tsunami that occurred in Chile on February 27, 2010, were selected. Structural equation modeling was used to analyze the data. The resulting model had adequate indices of goodness adjustment and showed that brooding completely mediated the relation between subjective severity and PTS, and DR completely mediated the relation between subjective severity, brooding, and PTG. These results highlight the role of both the content and process of rumination in mediating the association between subjective severity of trauma, PTS, and PTG. The implications of these results for a more comprehensive model of symptom severity that occurs after trauma are discussed.

One of the most devastating natural disasters that have affected South America in recent years was the earthquake and tsunami that occurred in Chile on February 27, 2010, which resulted in 521 deaths and 56 disappearances in addition to massive damages in infrastructure (Ministerio Público, 2011). A catastrophic event of this magnitude has significant short- and long-term psychological consequences (García, 2011). Studies that have explored the etiology of psychopathological problems such as depression, distress, and posttraumatic stress disorder (PTSD) after the occurrence of a natural disaster have often analyzed the impact of objective and subjective factors. On the one hand, there is the strong influence of the severity of objective factors or upheavals of life directly brought on by the event, such as the loss of one's home or non-negotiable and prolonged displacement (Dewaraja & Kawamura, 2006; Irmansyah, Dharmono, Maramis, & Minas, 2010; Sumer, Karanci, Berument, & Gunes, 2005). On the other hand, subjective severity or perceived risk, such as the fear of being wounded during a traumatic event or its meaning, also plays a role in the onset and maintenance of psychopathological symptoms (Lommen, Sanders, Buck, & Arntz, 2009; Roussos et al., 2005; Tural et al., 2001; Wahlström, Michélsen, Schulman, & Backheden, 2008; Xu & Song, 2011).

Subjective severity attributed to a traumatic experience has been shown to be a better predictor of posttraumatic stress symptomatology compared to factors related to the objective severity of a traumatic event (Boals & Schuettler, 2009; Morris, Shakespeare-Finch, Rieck, & Newberry, 2005). However, the relation between subjective severity and symptom severity is not necessarily direct. Janoff-Bulman (1992) proposed that catastrophic events may become psychologically traumatic to the extent that they shatter survivors’ basic beliefs in perceptions of personal worth, trust in others, and sense of world justice or predictability. According to this theory, shattering these beliefs could be the main factor through which psychological mechanisms are triggered that lead to either a reconstruction process of such beliefs or psychological distress (Arnoso et al., 2011). However, despite the plausibility of this model, very few studies have been conducted to empirically validate it (Bonanno, Westphal, & Mancini, 2011; Vázquez, 2013).

Further evidence of these complex relations is the fact that subjective severity of the trauma has also showed a positive direct relation with another consequence of the potentially traumatic events, which has been subject of study in recent years: posttraumatic growth (PTG García, Reyes, & Cova, 2014). PTG is defined as the perception of positive changes that occur as a result of the struggle against a trauma (Tedeschi & Calhoun, 1996). Growth and posttraumatic symptoms are not mutually exclusive. In fact, it is natural that they coexist somehow as they share as a common factor the exposure to a traumatic situation. However, there are indicators in order that long-term trajectories of people who develop a PTG are different from those who do not. More specifically, people who show PTG present greater well-being and less posttraumatic symptoms (Shakespeare-Finch & Lurie-Beck, 2014) than similar participants without PTG. Hence, the importance of identifying the specific factors that may help to predict one or another response.

Several motivational and cognitive mechanisms that can contribute to different, and sometimes opposite, psychological outcomes (e.g., meaning reconstruction or distress) after trauma and loss have been proposed (Park, 2010). One mechanism that has not received much attention but could be relevant in discussing the proposed relation between severity of a traumatic event and its associated psychological consequences (Weiss & Berger, 2010) is rumination. Rumination is defined as persistent and recurrent “thoughts that focus one's attention on one's depressive symptoms and on the implication of those symptoms” (Nolen-Hoeksema & Morrow, 1991, p. 569). Several authors have implicated rumination as a risk factor for the development of different psychopathologies. In a pioneer study of trauma, Nolen-Hoeksema and Morrow (1991) found that higher levels of rumination in a sample of students before the 1989 Loma Prieta (California) earthquake predicted greater symptoms of depression and PTSD 7 and 10 days after. This process has been shown to be transdiagnostically related to psychopathology, particularly with depression and anxiety states (Nolen-Hoeksema & Morrow, 1991; Papageorgiou & Wells, 2003; Walter & Bates, 2012).

It has been identified that brooding is the specific component of rumination that favors more negative emotional consequences (Boyraz & Efstathiou, 2011; Burwell & Shirk, 2007; Cova, Rincón, & Melipillán, 2009; Pimentel & Cova, 2011; Treynor, González, & Nolen-Hoeksema, 2003). Brooding is characterized by passive focus on the causes and consequences of negative emotions or experiences, recurrent comparison of the current situation with one that was not achieved, and dwelling on obstacles that prevent problems from being overcome (e.g., “What am I doing to deserve this?”; Treynor et al., 2003). Brooding has been associated with the persistence of PTSD up to 3 years after a traumatic event has occurred (Ehlers, Mayou, & Bryant, 1998, 2003) and with failure to achieve a therapeutic effect in cognitive–behavioral treatments of PTSD (Echiverri, Jaeger, Chen, Moore, & Zoellner, 2011). Brooding is also thought to mediate the association between traumatic experiences (such as childhood and sexual abuse) and depressive symptoms (Raes & Hermans, 2008). Moreover, Michael, Halligan, Clark, and Ehlers (2007) have demonstrated that brooding is responsible for triggering intrusive thoughts in PTSD. Collectively, these findings suggest that when a repetitive pattern of thoughts is maintained over a period of time, psychological distress does not dissipate (Cann et al., 2011; Morris & Shakespeare-Finch, 2011). Thus, brooding might be considered a vulnerability factor in the development and maintenance of PTSD (Kashdan, Young, & McKnight, 2012).

Another type of rumination that has been proposed refers to repetitive thoughts that are less automated and involves more deliberate and conscious efforts focused on handling that event, which has been called deliberate rumination (DR; Calhoun, Cann, Tedeschi, & McMillan, 2000). A study conducted in a sample of the general population in Madrid after a massive terrorist attack in 2004 provides indirect evidence of the importance of the nature of these different thought patterns (Vázquez, Hervás, & Pérez-Sales, 2008). In another study, a direct relation between DR and PTG has recently been observed. A study on Chilean adolescents who were exposed to the 2010 earthquake found that the association between subjective severity and PTG was partially mediated by DR (García, Jaramillo, Martínez, Valenzuela, & Cova, 2014). In contrast, the majority of studies have found no significant relations between DR and distress or PTSD (Morris & Shakespeare-Finch, 2011; Stockton, Hunt, & Joseph, 2011; Taku, Calhoun, Cann, & Tedeschi, 2008), with the exception of a study by Triplett et al. (2012) that found a significant, albeit small, effect of DR on PTSD.

In a cognitive processing model of trauma, cognitions and flashbacks or even intrusive images of the events experienced are natural and common reactions that occur often soon after a traumatic event (Taku, Cann, Tedeschi, & Calhoun, 2009). The persistence of a repetitive negative processing (brooding) can, however, contribute to the development and maintenance of negative emotional consequences. On the contrary, when the evolution of the reaction is positive, brooding can be replaced by a more deliberate style of rumination that facilitates the reconstruction of core assumptions about the world that have been shattered. Triplett et al. (2012) observed that both PTG and increases in life satisfaction appear only if brooding becomes DR, although distress might still persist.

This study aimed at evaluating a model that describes the effects of the subjective severity of trauma, brooding, and DR on posttraumatic symptoms (PTS) and PTG that develop in the aftermath of a potentially traumatic event. The model was analyzed using data gathered from individuals who lost their homes and many personal belonging as a result of the earthquake and tsunami in Chile in 2010. Based on the existing literature, we assessed whether the subjective severity of an event impacts PTS and PTG through indirect mediation of cognitive strategies (brooding and DR) rather than through direct means. We expected that brooding would mediate the relationship between subject severity and PTS, while a more elaborate type of cognition, deliberative rumination, would act as a mediator to PTG.

## 1. Materials and methods

### 1.1. Participants

The sample consisted of 351 individuals (63.2% female) who lost their homes as a result of the earthquake and tsunami that affected the Chilean Biobío Region on February 27, 2010, at 03:34 am. The earthquake reached a magnitude of 8.8 Mw and was one of eight more intense seismic events recorded in history (United States Geological Survey, 2010). Over 500,000 homes were severely damaged and nearly 2 million people were affected (Leiva, 2011). At the time of the study, participants lived in shelters (22.2%), rental homes (16%), homes provided by the Chilean government or purchased with a mortgage (40.2%), and with close friends or other families (20.8%). Due to a lack of reliable census of affected people, all participants were approached individually by the research team rather than through government organizations or NGOs. Participants ranged in age from 18 to 84 years (*M=*40.4, SD=15.29).

### 1.2. Procedure

Questionnaires were administered by psychologists and trained psychology students. Intentional sampling procedures were used to fulfill the target criterion of selecting people who lost their homes in the region of Biobío, an area of 37068.7 km^2^ with 1,971,998 inhabitants. The sampling sought that people who lost their homes for different causes were represented.

To achieve this, organized communities were visited and in the case of collapsed buildings and transitional shelters and fishing villages for tsunami-affected were visited; in the case of people who suffered irreparable damage in their homes the snowball technique was used. All participants signed an informed consent form before information was gathered. Data collection took place between September and November 2012.

### 1.3. Measurements

#### 1.3.1. Subjective severity

Two questions were used to assess this variable: “To what degree do you feel your life was disrupted as a result of the earthquake and/or tsunami?” and “To what degree do you rate the earthquake and/or tsunami as a traumatic experience for your life?” These items were each scored using a 5-point scale (ranging from 0=nothing to 4=severe) and were moderately correlated (*r*=0.64).

#### 1.3.2. Deliberate rumination

The DR subscale of Event Related Rumination Inventory (ERRI) was used (Cann et al., 2011). This questionnaire asks participants to rate the frequency of the occurrence of certain repetitive thoughts that deal with reflective aspects of the traumatic experience and its consequences (“I thought about whether I have learnt anything as a result of my experience,” “I thought about whether the experience has changed my beliefs about the world,” “I thought about what the experience might mean for my future”, and “I thought about whether my relationships with others have changed following my experience”) in the last 6 months, by responding on a 4-point Likert scale (0=not at all to 3=often). Its authors found a high internal consistency (*α*=0.88). García, Jaramillo, et al. (2014) obtained a Cronbach's alpha of 0.80, selecting for analysis only four items, because the remaining items were saturated in both factors, not saturated in either factor, or had crossed saturations. In the current study, these four items were utilized as indicators.

#### 1.3.3. Brooding

The brooding subscale from the Ruminative Response Scale by Treynor et al. (2003) was utilized. This five-item subscale consists of items reflecting a repetitive negative thinking style (e.g., “Why can't I handle things better?” and “What am I doing to deserve this?”) and was adapted to Spanish by Cova, Rincón, and Melipillan (2007). Later, two additional items were included (Cova et al., 2009). The scale used in the current study comprised seven items, each rated on a 4-point Likert scale ranging from 0 (almost never) to 3 (almost always) in relation to the frequency of ruminative negative thoughts in the first 6 months after the earthquake. The scale has good internal consistency, with a Cronbach's alpha of 0.80 (Cova et al., 2009).

#### 1.3.4. Posttraumatic symptoms

The Impact of the Event Scale-Revised (Weiss & Marmar, 1997) was utilized. This instrument was adapted for use in a Chilean population by Caamaño et al. (2011). The scale, covering the cluster of symptoms of PTSD according to the DSM-IV (1994), consists of eight items for Avoidance, six for Hyperarousal, and eight for Intrusion and assesses the severity of symptoms experienced over the past 6 days. Participants were asked to rank each item on a Likert scale ranging from 0 (nothing) to 4 (extremely). The scale has been shown to have good internal consistency (*α*=0.93; García, Jaramillo, et al., 2014). For the final analysis, three items were selected per factor (i.e., those that showed the highest factorial loads).

#### 1.3.5. Posttraumatic growth

The Posttraumatic Growth Inventory (PTGI) by Tedeschi and Calhoun (1996) was used, translated into Spanish by Páez et al. (2011). This instrument consists of 21 items that are scored on a Likert scale between 0 and 5 points. According to Tedeschi and Calhoun (1996), the internal consistency of the PTGI is high, *α*=0.91. This instrument has been previously adapted and validated for the Chilean population identifying three factors of improvement in life: Self-Perception (SP), Interpersonal Relationship (IR), and Life Philosophy (LP) (García, Cova, & Melipillán, 2013). In this study, Cronbach's alpha coefficients were high: *α*=0.97 for the total scale and 0.96 for SP, 0.93 for IR, and 0.87 for LP.

#### 1.3.6. Demographic information

A questionnaire was used to gather information on age, gender, city of residence at the time of the event, current city of residence, and cause of damage to the house.

### 1.4. Data analyses

Structural equation modeling (SEM) was carried out following the two-step procedure suggested by Anderson and Gerbing (1988). Confirmatory Factor Analysis was first carried out to assess the measurement model. This was then followed by the implementation of a structural model to test the hypothesis of the study. Lastly, the hypothesized model was compared to an alternative model to decide which one showed better fit, in concordance with previous recommendations by Martens (2005).

To estimate latent variables, individual items corresponding to the following variables were used: subjective severity (two items) and DR (four items). The seven items that comprised brooding were included in three parcels, as this strategy has been shown to improve the analyses by reducing the number of estimation parameters (Russell, Kahn, Spoth, & Altmaier, 1998). To create the parcels, we followed guidelines outlined by Russell et al. (1998) which required completion of an Exploratory Factor Analysis. This technique orders items according to their factor weights. Items are then assigned to each parcel in such a way that ensures their weights are balanced. In PTS and PTG, factors of the scale were maintained as factor indicators, and scores from the three individual items selected for each factor were added. The use of two different indicators in subjective severity to create a latent variable could be problematic in identifying a model. However, all criteria proposed by Ullman (2007) were followed to identify a robust model: (1) errors associated with the indicators were not correlated, (2) each indicator only loaded one factor, (3) factors were allowed to co-vary, and (4) latent variables with less than three indicators were associated with other variables (Yuan & Bentler, 2007).

Prior to the assessment of the measurement model, it was necessary to confirm that the indicators chosen to represent each construct were the most appropriate. To facilitate this, the composite reliability (CR) (Werts, Linn, & Jöreskog, 1974) and the average variance extracted (AVE; Fornell & Lacker, 1981) were used. Lèvy, Martín, and Román (2006) recommended that the CR must exceed a value of 0.7 and that the AVE should be greater than 0.5 for the construct to be considered as having convergent validity. For discriminant validity, the square root of the AVE (√AVE) of each latent variable must be greater than the correlation of all other latent variables. Otherwise, there is a possibility that the constructs are not conceptually different or that the indicators poorly differentiate the proposed constructs (Chin, 2010).

To evaluate the fit of the model, the Satorra-Bentler's mean adjusted maximum likelihood, a robust method for multivariate non-normality was used (Muthén & Muthén, 2010). The recommended strategy of relying on several fitting indices was followed (Marsh, Balla, & McDonald, 1988), because the “chi-square” is sensitive to sample size. The following criteria (Hu & Bentler, 1999; Yu, 2002) were utilized: (1) *χ* ^2^: a perfect fit is indicated by a non-significant value, (2) *χ* ^2^/gl: a good fit is indicated by a value lower than 2, (3) CFI and TLI: an acceptable fit is indicated by a value ≥0.90, whereas a good fit is indicated by a value ≥0.95, (4) PNFI: an acceptable fit is indicated by a value ≥0.50. (5) RMSEA: an acceptable fit is indicated by a RMSEA value ≤0.08 (90% CI≤0.10), whereas a good fit is indicated by a RMSEA ≤0.05 (90% CI≤0.08). PASW Statistics 18 and Mplus 7 software packages were used in this study.

## 2. Results

### 2.1. Reliability and validity of instruments

Analyses showed that all instruments had CR values that were greater than 0.87 and AVE values greater than 0.57, indicating that the constructs were internally consistent and had adequate convergent validity to proceed with testing of the model (Lèvy et al., 2006). Results from √AVE indicated that all constructs showed suitable discriminant validity. Table 1 presents all means, standard deviations, CR, AVE, √AVE, and relationships among the different measurements included in our study.

**Table 1:** Means, standard deviations, composite reliability (CR), AVE, √AVE, and correlations between main study measures

Measures	Min.	Max.	Mean	SD	CR	AVE	√AVE	2	3	4	5
SS	0	8	5.58	1.99	0.90	0.82	0.91	0.49***	0.43***	0.50***	0.32***
Brooding	0	21	8.95	5.73	0.90	0.57	0.76	–	0.54***	0.73***	0.28***
DR	0	12	6.21	3.19	0.88	0.64	0.80		–	0.55	0.44***
PTS	0	36	10.76	9.60	0.93	0.68	0.83			–	0.25***
PTG	0	45	29.22	11.19	0.93	0.61	0.78				–

SS, subjective severity; DR, deliberate rumination; PTS, posttraumatic symptoms; PTG, posttraumatic growth.

*** *p*<0.001.

### 2.2. Measurement model

After the selection of the indicators was finalized, they were gathered in parcels according to the previously detailed model (Table 2). This model yielded suitable fit indices *χ* ^2^(80)=106.550, *p>*0.05, *χ* ^2^/gl=1.331, CFI=0.99, TLI=0.99, RMSEA=0.031 (90% CI 0.01–0.05).

**Table 2:** Standardized factor loadings for latent variables in the measurement model

Latent variable	Factor indicators	SE	*Z*	Factor loadings
Subjective severity	S1	–	–	0.71
	S2	0.160	9.00	0.90
Brooding	BPAR1	–	–	0.86
	BPAR2	0.031	21.27	0.84
	BPAR3	0.033	19.61	0.88
Deliberate rumination	RUM1	–	–	0.73
	RUM2	0.088	12.46	0.75
	RUM3	0.092	13.03	0.79
	RUM4	0.089	10.64	0.63
Posttraumatic symptoms	Avoidance	–	–	0.89
	Hyperarousal	0.044	23.16	0.87
	Intrusion	0.038	26.13	0.93
Posttraumatic growth	Self-perception	–	–	0.87
	Interpersonal relations	0.081	16.99	0.89
	Life philosophy	0.048	12.99	0.65

All parameter estimates are significant at *p <*0.001.

### 2.3. Structural model for testing mediated effects

**Fig. 1:**
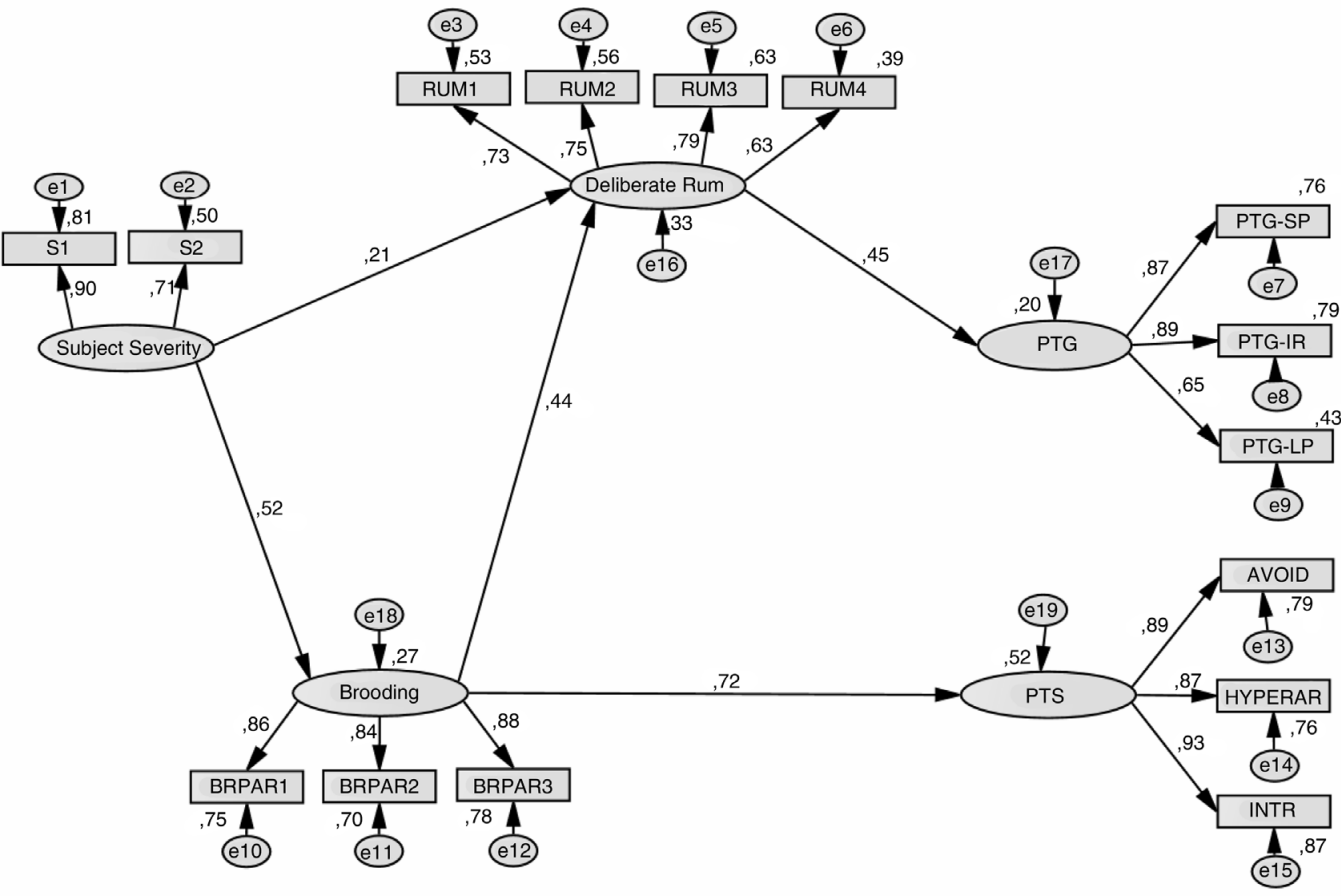
Hypothesized model 1. (See variable names in Table 2).

**Fig. 2:**
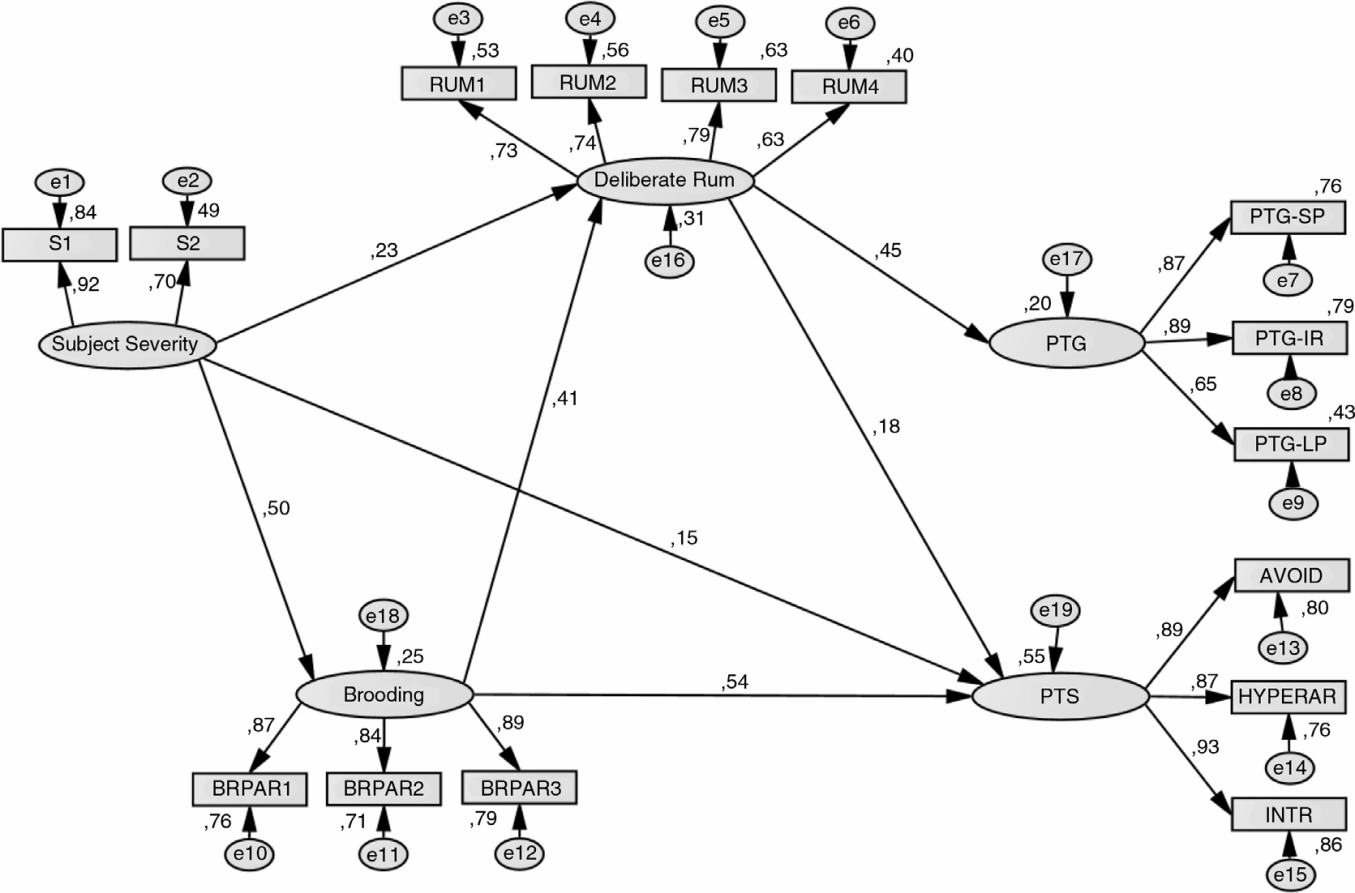
Alternative model 2 (See variable names in Table 2).

The hypothesized model shown in Fig. 1 (model 1) was evaluated. An alternative model (model 2) that proposed a direct effect between subjective severity and PTS (Morris & Shakespeare-Finch, 2011) and between DR and PTS (Triplett et al., 2012) was also evaluated (see Fig. 2). Model 2 presented suitable fit indices, its values were slightly higher compared with those in model 1, and the two added paths were significant but low weight, also loses in parsimony (PNFI). Thus, model 1 appeared to be the most appropriate for explaining the relationships among variables. Goodness-of-fit indicators for each model is shown in Table 3.

**Table 3:** Fit indices for model 1 and model 2

Model	*χ* ^2^ (gl)	*p*	*χ* ^2^/gl	CFI	TLI	PNFI	RMSEA (90% CI)
Model 1	169.97 (85)	0.00	2.00	0.97	0.97	0.77	0.053 (0.04–0.07)
Model 2	146.96 (83)	0.00	1.77	0.98	0.97	0.75	0.047 (0.03–0.06)

To analyze whether the indirect effects were significant, a bias-corrected bootstrap estimation (10,000 bootstrap samples with 95% confidence interval) was performed. Since zero was not included in any confidence interval, mediation was supported (MacKinnon, Lockwood, & Williams, 2004). Thus, bootstrapping analyses supported the hypothesis that DR completely mediates the relationship between subjective severity and brooding with PTG, and brooding completely mediates the relationship between subjective severity and PTS (these results are shown in Table 4). Furthermore, brooding showed similar moderate correlations with each cluster of the PTS symptoms: *r*=0.61 (Hyperarousal), *r*=0.59 (Avoidance), and *r*=0.61 (Intrusion).

**Table 4:** The standardized indirect effects, the 95% CI for the estimates (lower and upper bound), their standard errors, and *p*-values

Variable	Indirect effects (95% CI)	SE	*p*
SS→PTG	0.559/1.400	0.931	0.001
Brooding→PTG	0.185/0.432	0.291	0.001
SS→PTS	1.211/2.039	1.59	0.001

SS, subjective severity; PTG, posttraumatic growth; PTS, posttraumatic symptoms.

## 3. Discussion

The current study was performed to test a model of whether the subjective severity of an event impacts PTS and PTG through indirect mediation of cognitive strategies (brooding and DR) rather than through direct means. The resulting model showed adequate goodness-of-fit indices, thus supporting the initial hypotheses. The overall model suggested that while perceived severity of trauma makes an impact on PTS, this effect is completely mediated by cognitive strategies such as brooding; furthermore, DR fully mediate the relationship between subjective severity, brooding, and PTG. Our study shows that subjective severity alone is not sufficient to fully explain PTS or PTG, as its weight is low when cognitive variables related to elaborative processes are taken into account.

This finding is congruent with the theory by Janoff-Bulman (1992), according to which shattered schemas by a traumatic event can be ultimately reconstructed via elaborative cognitive processes (Janoff-Bulman, 2006), a process that resembles that of DR. Tedeschi and Calhoun (2004) argued that rumination occurs when the novel information produced by a traumatic event is incompatible with previously held core beliefs about security and predictability of the world. Therefore, it is possible that brooding could be followed by more intentional or elaborative cognitive processes, such as DR, in an attempt to rebuild basic assumptions (Calhoun & Tedeschi, 1998) and to generate hypotheses that are more adaptable to a new reality that incorporates the traumatic experience (Tedeschi & Calhoun, 2004; Tedeschi & Kilmer, 2005; Park, 2010). On the other hand, Moussa (2010) postulates that the presence of negative thoughts about a traumatic experience can be harmful, especially when they fail to lead to more deliberate thought processes or when those thoughts are perceived only as stressors. Therefore, brooding can be part of the usual and normal responses to highly stressful events, as well as other cognitive processes more or less automatic and intrusive. Indeed, brooding can contribute positively to more positive subsequent processing. However, this thinking mechanism can only have a beneficial role if an individual is pushed to the next stage of cognitive processing which attempts to integrate the traumatic experience into new concepts about the world. This can explain the direct relation of brooding with a negative consequence (PTS) as well as its direct relation with DR which, in turn, is related to a positive consequence (PTG).

Our findings on the importance of rumination are congruent with evidence showing that brooding mediates the appearance of maladaptive patterns of autobiographical memories (Romero, Vázquez, & Sánchez, 2014), in particular, traumatic memories (Brewin, 1989). In fact, Brewin model on the dual representation of traumatic recollections suggested that intrusion, a component of PTS, would be related to all of the contents stored in situationally accessible memory that had not yet been processed and integrated into structures of previous memories. Therefore, DR would be related to the adaptive role that the author proposes for the integration of these memories into verbally accessible memory (Brewin, 1989, 2001; Brewin, Dalgleish, & Joseph, 1996). Most studies (with the exception of the one by Triplett et al., 2012) have found that although DR is not associated with PTS, it might be related to adaptive outcomes (Benetato, 2011; García, Jaramillo, et al., 2014; Morris & Shakespeare-Finch, 2011; Stockton et al., 2011; Taku et al., 2008, 2009; Triplett et al., 2012).

One limitation of this study was that it was carried out 2 years after the earthquake; therefore, the results reported here could be different from those that might have been obtained immediately after the event. However, recall biases were minimized as participants were asked to report on current rather than retrospective symptoms of distress. Previous studies have relied on similar or even longer retrospective time intervals to assess the impact of traumatic events (Norris et al., 2002). A second limitation was that the data gathered were cross-sectional and correlational, although causal and temporary relationships were hypothesized through SEM. Therefore, the final model obtained, which suggests causal relationships between variables, must be confirmed with longitudinal and experimental methodologies in future studies.

Along with overcoming these limitations, future research that incorporates elements related to the process of emotional regulation rather than aspects solely related to the traumatic event would be of interest. For instance, recent evidence has shown that emotional overproduction (i.e., the coexistence of many different negative emotions) is a critical antecedent of rumination (Hervás & Vázquez, 2011). Factors that influence the transformation of brooding into DR and the role of the latter in adaptive processes or PTG should also be identified.

## Supplementary Material

Trauma or growth after a natural disaster? The mediating role of rumination processes

Trauma or growth after a natural disaster? The mediating role of rumination processes

Trauma or growth after a natural disaster? The mediating role of rumination processes

Trauma or growth after a natural disaster? The mediating role of rumination processes

Trauma or growth after a natural disaster? The mediating role of rumination processes
