# Supplementary material for: Trauma or growth after a natural disaster? The mediating role of rumination processes
Source: Eur J Psychotraumatol. 2015 Jul 31;6:10.3402/ejpt.v6.26557. doi: 10.3402/ejpt.v6.26557 (PMC4522433; doi:10.3402/ejpt.v6.26557)
Supplement: Trauma or growth after a natural disaster? The mediating role of rumination processes [file EJPT-6-26557-s001.pdf]

Title: ¿Trauma o crecimiento después de un desastre natural? El rol mediador de los procesos de ruminación

Felipe E. García, Félix Cova, Paulina Rincón, Carmelo Vázquez

El objetivo de este estudio es probar un modelo cognitivo de síntomas postraumáticos (SPT) y crecimiento postraumático (CPT) después de la exposición a un desastre natural. Se planteó la hipótesis de que aunque la realidad subjetiva del trauma estaría relacionada con la gravedad de los SPT, esta relación estaría mediada por estrategias cognitivas y depresivas relacionadas con la presencia de un contenido negativo repetitivo en el pensamiento. Es más, la relación entre la gravedad y el CPT estaría mediada completamente por la ruminación deliberada, estrategias cognitivas relacionadas con esfuerzos conscientes centrados en manejar el acontecimiento. Para evaluar el modelo cognitivo, se seleccionaron adultos (N = 351) que habían perdido sus hogares como resultado del terremoto y el tsunami de Chile el 27 de febrero de 2010. Para analizar los datos se utilizó el modelo de ecuación estructural. El modelo resultante tenía unos índices adecuados de bondad de ajuste y mostraba que la melancolía mediaba por completo en la relación entre la gravedad subjetiva y los SPT y que la ruminación deliberada mediaba completamente en la relación entre la gravedad subjetiva, la melancolía y el CPT. Estos resultados resaltan el papel tanto del contenido como del proceso de ruminación a la hora de intervenir en la asociación entre la gravedad subjetiva, los SPT y el CPT. Se debaten las mplicaciones de estos resultados para un modelo más exhaustivo de la gravedad de los síntomas que ocurren después de un trauma.

Palabras clave: Búsqueda de beneficios; angustia; terremoto; desastre natural; rumínación; síntomas de estrés postraumático; tsunami

Name of translator: Miriam Ramos Morrison

Citation: European Journal of Psychotraumatology 2015, **6**: 26557 - http://dx.doi.org/10.3402/ejpt.v6.26557
