# Supplementary material for: Trauma or growth after a natural disaster? The mediating role of rumination processes
Source: Eur J Psychotraumatol. 2015 Jul 31;6:10.3402/ejpt.v6.26557. doi: 10.3402/ejpt.v6.26557 (PMC4522433; doi:10.3402/ejpt.v6.26557)
Supplement: Trauma or growth after a natural disaster? The mediating role of rumination processes [file EJPT-6-26557-s002.pdf]

Trauma or growth after a natural disaster? The mediating role of rumination processes

Felipe E. García, Félix Cova, Paulina Rincón, Carmelo Vázquez

Celem niniejszej pracy było przetestowanie poznawczego modelu objawów potraumatycznych i potraumatycznego wzrostu w konsekwencji doświadczenia katastrofy naturalnej. Założono, że choć subiektywny poziom ekspozycji na traumę będzie związany z natężeniem objawów potraumatycznych, to jednak subiektywny poziom ekspozycji na traumę będzie mediowany przez ruminacyjne i poznawcze strategie związane z obecnością negatywnych treści myslowych. Dodatkowo założyliśmy, że związek pomiędzy subiektwną ekspozycją na traumę oraz poziomem potraumatycznego wzrostu będzie całkowicie mediowany przez ruminacje deliberatywne oraz poznawcze strategie radzenia sobie z traumatycznym zdarzeniem. W badaniu wzięła udział grupa 351 ofiar trzęsienia ziemi w Chile w 2010 roku. Modelowanie równań strukturalnych wykazało, że zaproponowany przez nas model jest dobrze dopasowany do danych. Model ten wykazał, że ruminacje całkowicie mediowały relację pomiędzy subiektywną ekspozycją na traumę, poziomem objawów PTSD oraz natężeniem potraumatycznego wzrostu.

Słowa kluczowe: poszukiwanie korzyści; dystres; trzęsienie ziemi; katastrofa naturalna, ruminacje; objawy potraumatyczne; tsunami

Name of translator: Marcin Rzeszutek, University of Finance and Management in Warsaw, Poland

Citation: European Journal of Psychotraumatology 2015, 6: 26557 - http://dx.doi.org/10.3402/ejpt.v6.26557
