# Supplementary material for: Trauma or growth after a natural disaster? The mediating role of rumination processes
Source: Eur J Psychotraumatol. 2015 Jul 31;6:10.3402/ejpt.v6.26557. doi: 10.3402/ejpt.v6.26557 (PMC4522433; doi:10.3402/ejpt.v6.26557)

Accepted but unedited

¿Trauma o crecimiento después de un desastre natural? El rol mediador de los procesos de rumiación

Felipe E. García[1, 2]

Félix Cova Solar[3]

Paulina Rincón González[4]

Carmelo Vázquez[5]

---

[1] Correspondencia a: Escuela de Psicología, Universidad Santo Tomás, Chile. Av. Prat 855, Concepción (Chile). Correo electrónico: felipegarciam@yahoo.es

[2] Programa de Doctorado en Psicología, Universidad de Concepción, Chile

[3] Facultad de Psicología, Universidad de Concepción, Chile.

[4] Facultad de Psicología, Universidad de Concepción, Chile.

[5] Facultad de Psicología, Universidad Complutense, Madrid, España.

¿Trauma o crecimiento después de un desastre natural? El rol mediador de los procesos de rumiación

Resumen

El objetivo de este estudio fue probar un modelo cognitivo de sintomatología postraumática (SPT) y de crecimiento postraumático (CPT) tras la exposición a un desastre natural. Se hipotetizó que aunque la severidad subjetiva del evento estaría relacionada con la severidad de la SPT, esta relación estaría mediada por la rumiación negativa, una estrategia cognitiva relacionada con la presencia de pensamientos negativos repetitivos. Además, la relación entre la severidad y el CPT estaría completamente mediada por la rumiación deliberada, estrategia cognitiva relacionada con los esfuerzos conscientes enfocados en el manejo del evento. Para evaluar el modelo cognitivo, se seleccionaron adultos (N = 531) que perdieron sus casas como resultado del terremoto y tsunami que ocurrió en Chile el 27 de febrero del 2010. Para analizar los datos se utilizó un modelo de ecuaciones estructurales. El modelo resultante tuvo índices adecuados de bondad de ajuste y mostró que la rumiación negativa mediaba completamente la relación entre la severidad subjetiva y SPT y que la rumiación deliberada mediaba completamente la relación entre la severidad subjetiva, la rumiación negativa y el CPT. Estos resultados recalcan el rol tanto del contenido como del proceso de rumiación en la mediación de la asociación entre la severidad subjetiva del trauma, la SPT y el CPT. Se discuten las implicancias de estos resultados para un modelo más completo de la severidad de los síntomas que ocurren después de un trauma.

Palabras claves: percepción de beneficios; angustia; terremoto; desastre natural; rumiación; síntomas de estrés postraumático; tsunami

1. Introducción

Uno de los desastres naturales más devastadores que ha afectado a Sudamérica en los últimos años fue el terremoto y tsunami ocurrido en Chile el 27 de febrero del 2010, que tuvo como resultado 521 muertos y 56 desaparecidos además de los enormes daños a la infraestructura (Ministerio Público, 2011). Una catástrofe de esta magnitud tiene consecuencias psicológicas significativas tanto a corto como a largo plazo (García, 2011). Los estudios que han explorado la etiología de los problemas psicopatológicos tales como la depresión, la angustia y el trastorno por estrés post-traumático (TEPT) después de la ocurrencia de un desastre natural generalmente han analizado el impacto de los factores objetivos y subjetivos. Por un lado, existe una fuerte influencia de la severidad objetiva o de las alteraciones efectivas en la vida provocadas directamente por el evento, tales como la pérdida de hogar o el desplazamiento forzado y prolongado (Dewaraja & Kawamura, 2006; Irmansyah, Dharmono, Maramis, & Minas, 2010; Sumer, Karanci, Berument, & Gunes, 2005). Por otro lado, la severidad subjetiva o el riesgo percibido, tales como el miedo a ser lastimado durante un evento traumático o su significado, también juega un rol en el inicio y la mantención de los síntomas psicopatológicos (Lommen, Sanders, Buck, & Arntz, 2009; Roussos et al., 2005; Tural et al., 2001; Wahlström, Michélsen, Schulman, & Backheden, 2008; Xu & Song, 2011).

Se ha demostrado que la severidad subjetiva atribuida a una experiencia traumática es un mejor predictor de la sintomatología postraumática (SPT) comparada con los factores relacionados con la severidad objetiva de un evento traumático (Boals & Schuettler, 2009; Morris, Shakespeare-Finch, Rieck, & Newberry, 2005). Sin embargo, la relación entre la severidad subjetiva y la severidad de la SPT no es necesariamente directa. Janoff-Bulman (1992) propuso que los eventos catastróficos pueden volverse traumáticos psicológicamente en la medida que ellos destruyen las

creencias básicas de los sobrevivientes sobre las percepciones de la valía personal, la confianza en los otros y el sentido de justicia y previsibilidad del mundo. De acuerdo a esta teoría, la destrucción de estas creencias sería el principal factor a través del cual se gatillan los mecanismos psicológicos que llevan ya sea a un proceso de reconstrucción de tales creencias o a la angustia psicológica (Arnoso et al., 2012). Sin embargo, a pesar de la verosimilitud de este modelo, se han realizado muy pocos estudios para validarlo empíricamente (Bonanno et al., 2011; Vázquez, 2013).

Una evidencia más de estas relaciones complejas es el hecho de que la severidad subjetiva del trauma también ha mostrado una relación positiva con otra consecuencia de los eventos potencialmente traumáticos que ha sido sujeto de estudio en los últimos años: el crecimiento post-traumático (CPT; García, Reyes, & Cova, 2014). El CPT se define como la percepción de cambios positivos que ocurre como resultado de la lucha contra un trauma (Tedeschi & Calhoun, 1996). El CPT y la SPT no son excluyentes. De hecho, es natural que ellos coexistan de algún modo ya que comparten en común la exposición a una situación traumática. Sin embargo, existe evidencia de que las trayectorias a largo plazo de las personas que desarrollan CPT son diferentes a las de aquellas que no lo hacen. De manera más específica, las personas que muestran CPT presentan mayor bienestar y menos SPT (Shakespeare-Finch & Lurie-Beck, 2014) que los participantes similares sin CPT. De ahí, la importancia de la identificación de los factores específicos que pueden ayudar a predecir una respuesta u otra.

Se han propuesto varios mecanismos motivacionales y cognitivos que pueden contribuir a resultados psicológicos diferentes y, a veces, opuestos (ej.: es decir, reconstrucción o angustia) después del trauma y la pérdida (Park, 2010). Un mecanismo que no ha recibido mucha atención pero que podría ser relevante al discutir la relación propuesta entre la severidad de un evento y sus consecuencias psicológicas asociadas

(Weiss & Berger, 2010) es la rumiación, definida como los "pensamientos persistentes y recurrentes que enfocan la atención en los síntomas depresivos y en la implicancia de estos síntomas" (Nolen-Hoeksema & Morrow, 1991, p. 569). Varios autores toman la rumiación implícita como un factor de riesgo para el desarrollo de las diferentes psicopatologías. En un estudio pionero sobre el trauma, Nolen-Hoeksema y Morrow (1991) encontraron que los niveles más altos de rumiación en una muestra de estudiantes antes del terremoto de Loma Prieta (California) en 1989 predijeron mayores síntomas de depresión y TEPT a los diez días y siete semanas después. Se ha demostrado que este proceso tiene una relación transdiagnóstica con la psicopatología, particularmente con los estados de depresión y ansiedad (Nolen-Hoeksema & Morrow, 1991; Papageorgiou & Wells, 2003; Walter & Bates, 2012).

Se ha identificado que la rumiación negativa o brooding es el componente específico de la rumiación que favorece las consecuencias emocionales más negativas (Boyraz & Efstathiou, 2011; Burwell & Shirk, 2007; Cova, Rincón, & Melipillán, 2009; Pimentel & Cova, 2011; Treynor, González, & Nolen-Hoeksema, 2003). La rumiación negativa se caracteriza por la focalización pasiva sobre las causas y consecuencias de las emociones o experiencias negativas, la comparación recurrente de la situación actual con una que no se logró y el pensamiento obsesivo sobre los obstáculos que evitan que los problemas sean superados (ej.: "*¿Qué estoy haciendo para merecer ésto?*") (Treynor et al., 2003). Se ha asociado a la rumiación negativa con la persistencia del TEPT hasta tres años después de que ha ocurrido un evento traumático (Ehlers, Mayou, & Bryant, 1998, 2003) y con la falla en lograr un efecto terapéutico en los tratamientos cognitivos-conductuales del TEPT (Echiverri, Jaeger, Chen, Moore, & Zoellner 2011). También se piensa que la rumiación negativa media la asociación entre las experiencias traumáticas (tales como el abuso infantil y sexual) y los síntomas depresivos (Raes y

Hermans, 2008). Además, Michael, Halligan, Clark, y Ehlers (2007) han demostrado que la rumiación negativa es responsable de gatillar los pensamientos invasivos en los TEPT. De manera general, estos hallazgos sugieren que cuando se mantiene un patrón repetitivo de pensamientos por un tiempo, la angustia psicológica no se disipa (Cann et al., 2011; Morris & Shakespeare-Finch, 2011). Así, la rumiación negativa se podría considerar un factor de vulnerabilidad en el desarrollo y la mantención del TEPT (Kashdan, Young, & McKnight, 2012).

Otro tipo de rumiación que se ha propuesto es la llamada rumiación deliberada (RD), que se refiere a los pensamientos repetitivos que son menos automatizados e involucran esfuerzos más deliberados y conscientes enfocados en el manejo de ese evento (Calhoun, Cann, Tedeschi, y McMillan, 2000). Un estudio realizado en una muestra de la población general en Madrid después de un ataque terrorista masivo el 2004 entrega evidencia indirecta de la importancia de la naturaleza de estos diferentes patrones de pensamiento (Vázquez, Hervás, & Pérez-Sales, 2008). En otro estudio más reciente, realizado con adolescentes chilenos que estuvieron expuestos al terremoto del 2010, se ha observado una relación directa entre la RD y el CPT; específicamente se encontró que la asociación entre la severidad subjetiva y el CPT estaba mediada parcialmente por la RD (García, Jaramillo, Martínez, Valenzuela, & Cova, 2014). Por el contrario, la mayoría de los estudios no han encontrado relaciones significativas entre la RD y la angustia o el TEPT (Morris & Shakespeare-Finch, 2011; Stockton, Hunt, & Joseph, 2011; Taku et al., 2008), con la excepción de un estudio realizado por Triplett et al. (2012) que encontró un efecto significativo, aunque pequeño, de la RD sobre el TEPT.

En un modelo de procesamiento cognitivo del trauma, que se potencien cogniciones y flashbacks e incluso imágenes intrusivas de los eventos vividos, es una reacción natural e inevitable que ocurre poco después de un evento traumático (Taku,

Cann, Tedeschi, & Calhoun, 2009). La persistencia de este procesamiento repetitivo negativo (rumiación negativa) puede, sin embargo, contribuir al desarrollo y la mantención de las consecuencias emocionales negativas. Por el contrario, cuando la evolución de esta reacción es positiva, la rumiación negativa puede ser reemplazada por un estilo de rumiación más deliberado que facilitará la reconstrucción de supuestos fundamentales sobre el mundo que se han destruido. Triplett et al. (2012) observaron que tanto el CPT como una mayor satisfacción con la vida aparecen solo si la rumiación negativa se convierte en RD, aunque la angustia aún podría persistir.

Este estudio pretende evaluar un modelo que describe los efectos de la severidad subjetiva del trauma, la rumiación negativa y la RD sobre la SPT y el CPT que se desarrollan tras la exposición a un evento potencialmente traumático. El modelo se analizó utilizando los datos recolectados en personas que perdieron sus hogares y muchas pertenencias personales como resultado del terremoto y tsunami en Chile el 2010. Basándose en la literatura existente, se propuso un modelo inicial (ver Figura 1) en el que la severidad subjetiva impacta indirectamente en la SPT, mediada parcialmente por la rumiación negativa, e impacta indirectamente en el CPT mediada completamente por la RD. El modelo además sugiere que la RD media completamente la relación entre la rumiación negativa y el CPT. Finalmente, el modelo predice que la RD no está relacionada significativamente con la SPT.

2. Materiales y métodos

2.1. Participantes

La muestra consta de 351 personas (63,2 % mujeres) que perdieron sus hogares como resultado del terremoto y tsunami que afectó a la región chilena del Biobío el 27 de febrero del 2010 a las 03:34 am. El terremoto alcanzó una magnitud de 8,8 Mw y fue uno de los ocho eventos sísmicos más intensos registrados en la historia (United States

Geological Survey, 2010). Más de 500.000 hogares se vieron severamente dañados y casi dos millones de personas fueron afectadas (Leiva, 2011). Al momento del estudio, los participantes vivían en albergues (22,2%), casas arrendadas (16%), casas entregadas por el gobierno de Chile o compradas con un crédito hipotecario (40,2%) y con amigos cercanos o con otras familias (20,8%). Debido a la falta de un censo confiable de las personas afectadas, el equipo de investigación se acercó individualmente a todos los participantes más que hacerlo a través de organizaciones gubernamentales u ONGs. Los participantes variaban en edad desde los 18 a los 84 años (*M*=40,4, *DE*=15,29).

2.2.Procedimiento

Psicólogos y estudiantes de psicología capacitados administraron los cuestionarios. Se utilizaron procedimientos de muestreo intencionales para cumplir con el criterio objetivo de seleccionar a personas que perdieron sus hogares en la región del Biobío, un área de 37.068,7 km^2 con 1.971.998 habitantes. El muestreo buscó que en el grupo se representaran personas que perdieron sus hogares por diferentes causas.

Para lograr ésto, se visitaron comunidades organizadas en el caso de los edificios desplomados; se visitaron campamentos transitorios y caletas de pescadores para acceder a personas afectadas por el tsunami; en el caso de las personas que sufrieron daños irreparables en sus hogares, se utilizó la técnica de bola de nieve. Todos los participantes firmaron una carta de consentimiento informado antes de que se recolectara la información. La recolección de datos se realizó entre septiembre y noviembre del 2012.

2.3.Mediciones

2.3.1. Severidad subjetiva. Para evaluar esta variable se utilizaron dos preguntas: "*¿en qué grado usted siente que su vida se vio alterada producto del*

terremoto y/o tsunami?” y “*¿en qué grado califica usted el terremoto y/o tsunami como una experiencia traumática para su vida?*”. Cada uno de estos ítemes se calificaron utilizando una escala de 5 puntos (que va desde 0 = nada a 4 = severo) y se correlacionaron moderadamente (r = 0,64).

2.3.2. Rumiación deliberada. Se utilizó la subescala de rumiación deliberada del Inventario de Rumiación Relacionada al Evento (ERRI por su sigla en inglés) (Cann et al., 2011). Este cuestionario pide a los participantes clasificar la frecuencia de la ocurrencia de ciertos pensamientos repetitivos (ej.: “Pienso en si esta experiencia ha cambiado mi percepción del mundo”) en los últimos seis meses, respondiendo ésta en una escala de Likert de 4 puntos (0 = en lo absoluto a 3 = a menudo). Sus autores encontraron una consistencia interna alta (α=0,88). García, Jaramillo et al. (2014) obtuvieron un alfa de Cronbach de 0,80 seleccionando para el análisis solo cuatro ítemes ya que los ítemes restantes estaban saturados en ambos factores, no estaban saturados en ningún factor o tenían saturaciones cruzadas. En el presente estudio, se utilizaron esos 4 ítems como indicadores.

2.3.3. Rumiación negativa. Se utilizó la subescala de rumiación negativa de la Escala de Respuesta Rumiativa de Treynor et al. (2003), adaptada por Cova, Rincón y Melipillán al español (2007). Esta subescala de 5 ítems consta de afirmaciones tales como “Pienso acerca de una situación reciente, deseando que hubiera resultado mejor”. Luego, se incluyeron dos ítemes adicionales (Cova et al., 2009). La escala utilizada en el presente estudio estuvo formada por siete ítemes, cada uno clasificado en una escala de Likert de 4 puntos que va desde 0 (casi nunca) a 3 (casi siempre), en relación a la frecuencia de los pensamientos negativos rumiativos en los primeros seis meses tras el terremoto. La escala tuvo una buena consistencia interna con un alfa de Cronbach de 0,80 (Cova et al., 2009).

2.3.4. Sintomatología postraumática. Se utilizó la Escala de Impacto de Evento Revisada (IES-R por su sigla en inglés; Weiss & Marmar, 1997). Este instrumento fue adaptado por Caamaño et al. (2011) para su uso en la población chilena. La escala, que abarca el grupo de síntomas de los TEPT de acuerdo al DSM-IV (1994), consta de ocho ítemes para Evitación, seis para Hiperactivación y ocho para Intrusión y evalúa la severidad de los síntomas experimentados en los últimos seis días. Se le pidió a los participantes clasificar cada ítem en una escala de Likert que va desde 0 (nada) a 4 (extremadamente). Se ha demostrado que la escala tiene una adecuada consistencia interna (α=0,93) (García, Jaramillo et al., 2014). Para el análisis final se seleccionaron tres ítemes por factor (es decir, aquellos que mostraron las cargas factoriales más altas).

2.3.5. Crecimiento postraumático. Se utilizó el Inventario de Crecimiento Postraumático (PTGI por su sigla en inglés) de Tedeschi y Calhoun (1996) traducido al español por Páez et al. (2011). Este instrumento consta de 21 ítemes que se califican en una escala de Likert entre 0 y 5 puntos. De acuerdo a Tedeschi y Calhoun (1996), la consistencia interna del PTGI es alta, α=0,91. Este instrumento ha sido adaptado y validado previamente para la población chilena identificando tres factores: Auto-Percepción (AP), Relación Interpersonal (RI) y Filosofía de Vida (FV) (García, Cova, & Melipillán, 2013). En este estudio, los coeficientes alfa de Cronbach fueron altos: α=0,97 para la escala total y 0,96 para AP; 0,93 para RI y 0,87 para FV.

2.3.6. Información demográfica. Se utilizó un cuestionario para recolectar información sobre la edad, sexo, ciudad de residencia al momento del evento, ciudad actual de residencia y causa del daño a la casa.

2.4. Análisis de datos

El modelado de ecuaciones estructurales (SEM, por sus siglas en inglés) se realizó siguiendo el procedimiento de dos pasos sugerido por Anderson y Gerbing (1988). Primero se realizó un Análisis Factorial Confirmatorio para evaluar el modelo de medida. Luego, fue seguido por la evaluación de un modelo estructural para probar la hipótesis del estudio. Finalmente, el modelo hipotetizado se comparó con un modelo alternativo para decidir cuál mostraba mejor ajuste de acuerdo con las recomendaciones anteriores de Martens (2005).

Para estimar las variables latentes, se utilizaron ítems individuales en las variables de severidad subjetiva (dos ítemes) y rumiación deliberada (cuatro ítemes). Los siete ítemes que comprenden la rumiación negativa se agruparon en tres *parcels*, ya que se ha demostrado que esta estrategia mejora los análisis al reducir el número de parámetros de estimación (Russell, Kahn, Spoth, & Altmaier, 1998). Para crear los *parcels*, los investigadores siguieron las directrices descritas por Russell et al. (1998) que requerían completar un Análisis Factorial Exploratorio (AFE). Esta técnica ordena los ítemes de acuerdo a sus pesos factoriales. Luego, los ítemes son asignados a cada *parcel* de tal manera de asegurar que sus pesos estén balanceados. En la SPT y el CPT, se mantuvieron los factores de la escala como indicadores sumando los puntajes de los tres ítemes individuales seleccionados para cada factor. El uso de dos indicadores en la severidad subjetiva para crear la variable latente podría ser problemático para la identificación del modelo. Sin embargo, se siguieron todos los criterios propuestos por Ullman (2007) para identificar un modelo robusto: a) los errores asociados con los indicadores no están correlacionados, b) cada indicador carga solo en un factor, c) se permitió que los factores covaríen y d) las variables latentes con menos de tres indicadores están asociadas con otras variables (Yuan & Bentler, 2007).

Antes de la evaluación del modelo de medida, fue necesario confirmar que los indicadores elegidos para representar cada constructo eran los más apropiados. Para facilitar ésto, se utilizó la confiabilidad compuesta (Werts, Linn, & Jöreskog, 1974) y la varianza extraída promedio (AVE por su sigla en inglés; Fornell & Lacker, 1981). Para que se considere que el constructo tiene validez convergente, Lèvy, Martín y Román (2006) recomendaron que la confiabilidad compuesta debe exceder un valor de 0,7 y que la AVE debería ser mayor que 0,5. Para la validez discriminante, la raíz cuadrada de la AVE (√AVE) de cada variable latente debe ser mayor que la correlación de todas las otras variables latentes. De otro modo, existe una posibilidad de que los constructos no sean conceptualmente diferentes o de que los indicadores diferencien pobremente los constructos propuestos (Chin, 2010).

Para evaluar el ajuste del modelo, se utilizó como método de estimación la Máxima Verosimilitud Ajustada por la Media de Satorra-Bentler (MLM, por sus siglas en inglés), un método robusto para la no-normalidad multivariante (Muthén & Muthén, 2010). Se siguió la estrategia recomendada de depender de varios índices de ajuste (Marsh, Balla, & McDonald, 1988) ya que la "chi-cuadrada" es sensible al tamaño de la muestra. Se utilizaron los siguientes criterios (Hu & Bentler, 1999; Yu, 2002): a) χ^2: un valor no significativo indica un ajuste perfecto, b) χ^2/gl: un valor menor a 2 indica un buen ajuste, c) CFI y TLI: un valor ≥ 0,90 indica un ajuste aceptable mientras que un valor ≥ 0,95 indica un buen ajuste, c) PNFI: un valor ≥ 0,50 indica un ajuste aceptable, d) RMSEA: un valor RMSEA ≤ 0,08 (90% CI≤ 0,10) indica un ajuste aceptable mientras que un RMSEA ≤ 0,05 (90% CI≤ 0,08) indica un buen ajuste. En este estudio se utilizaron los paquetes de software PASW Statistics 18 y Mplus 7.

3. Resultados

3.1.Confiabilidad y validez de los instrumentos

Los análisis mostraron que todos los instrumentos tenían valores de confiabilidad compuesta mayores que 0,87 y valores AVE mayores que 0,57 lo que indica que los constructos eran internamente consistentes y que tenían una validez convergente adecuada para continuar con las pruebas del modelo (Lèvy et al., 2006). Los resultados de la √AVE indicaron que todos los constructos mostraron una validez discriminante adecuada. La Tabla 1 presenta todas las medias, las desviaciones estándares, la confiabilidad compuesta, la AVE, la √AVE y las relaciones entre las diferentes mediciones incluidas en nuestro estudio.

Insertar Tabla 1

3.2.Modelo de medida

Luego de terminada la selección de los indicadores, éstos se recolectaron en secciones de acuerdo al modelo detallado anteriormente (consultar Tabla 2). Este modelo produjo índices de ajuste adecuados $\chi^2(80)=106{,}550$; $p>0{,}05$; $\chi 2/gl=1{,}331$; CFI=0,99; TLI=0,99; RMSEA=0,031 (90% CI=0,01-0,05).

Insertar Tabla 2

3.3.Modelo estructural para probar los efectos mediados

Se evaluó el modelo hipotetizado mostrado en la Figura 1 (modelo 1). También se evaluó un modelo alternativo (modelo 2) que propuso un efecto directo entre la

severidad subjetiva y la SPT (Morris & Shakespeare-Finch, 2011) y entre la RD y la SPT (Triplett et al., 2012). El modelo 2 presentó índices de ajuste adecuados, sus valores fueron levemente más altos comparados con aquellos del modelo 1 y los dos caminos agregados eran significativos pero de bajo peso, además de pérdidas en la parsimonia (PNFI). Así, el modelo 1 parece ser el más apropiado para explicar las relaciones entre las variables. La bondad de los indicadores de ajuste para cada modelo se muestra en la Tabla 3.

Insertar Figura 1

Insertar Figura 2

Insertar Tabla 3

Para analizar si los efectos indirectos eran significativos, se realizó una estimación bootstrap de sesgo-corregido (10.000 muestras bootstrap con intervalo de confianza de 95%). En este caso, la mediación existe si el cero no está incluido en el intervalo de confianza. (MacKinnon, Lockwood & Williams, 2004). Así, los análisis con bootstrapping respaldaron la hipótesis de que la RD media completamente la relación entre la severidad subjetiva y la rumiación negativa con el CPT y que la rumiación negativa media completamente la relación entre la severidad subjetiva y la SPT (estos resultados se muestran en la

Tabla 4). Además, la rumiación negativa mostró correlaciones moderadas similares con cada grupo síntomas de la SPT: r=0,61, (Hiperactivación), r=0,59 (Evitación) y r=0,61 (Intrusión).

Insertar Tabla 4

----

4. Discusión

El presente estudio se realizó para probar un modelo en el que la severidad subjetiva de un evento impacta a la SPT y al CPT a través de la mediación indirecta de las estrategias cognitivas (rumiación negativa y RD) más que en forma directa. El modelo resultante mostró una adecuada bondad de los índices de ajuste respaldando así la hipótesis inicial. El modelo general sugirió que mientras la severidad percibida del evento tiene un impacto en la SPT, este efecto está mediado completamente por las estrategias cognitivas tales como la rumiación negativa; además, la RD media completamente la relación entre la severidad subjetiva, la rumiación negativa y el CPT. Nuestro estudio muestra que la severidad subjetiva sola no es suficiente para explicar completamente los SPT o el CPT ya que su peso es bajo cuando se toman en cuenta las variables cognitivas relacionadas con los procesos elaborativos.

Este hallazgo es congruente con la teoría de Janoff-Bulman (1992) según la cual los esquemas destruidos por un evento traumático en definitiva se pueden reconstruir a través de procesos cognitivos elaborativos (Janoff-Bulman, 2006), un proceso que se asemeja al de la RD. Tedeschi y Calhoun (2004) sostienen que la rumiación ocurre cuando la información nueva producida por un evento traumático es incompatible con las creencias fundamentales que se tenían previamente acerca de la seguridad y la previsibilidad del mundo. Por lo tanto, es posible que la rumiación negativa pudiese

estar seguida por procesos cognitivos más intencionales o elaborativos, tales como la RD, en un intento por reconstruir los supuestos básicos (Calhoun & Tedeschi, 1998) y generar hipótesis que son más adaptables a una nueva realidad que incorpora la experiencia traumática (Tedeschi & Calhoun, 2004; Tedeschi & Kilmer, 2005; Park, 2010). Por otro lado, Moussa (2010) postula que la presencia de pensamientos negativos sobre una experiencia traumática puede ser dañina, especialmente cuando falla en llevar a procesos de pensamientos más deliberados o cuando aquellos pensamientos solo se perciben como estresores. Por lo tanto, la rumiación negativa puede ser parte de las respuestas habituales y normales a eventos altamente estresantes así como también otros procesos cognitivos más o menos automáticos e invasivos. De hecho, la rumiación negativa puede contribuir positivamente a un procesamiento posterior más positivo. Sin embargo, este mecanismo de pensamiento solo puede tener un rol beneficioso si se empuja a un individuo a pasar a la siguiente etapa del procesamiento cognitivo que intenta integrar la experiencia traumática a nuevas concepciones acerca del mundo. Esto puede explicar la relación directa de la rumiación negativa con una consecuencia negativa (SPT) así como también su relación directa con la rumiación deliberada, la que a su vez está relacionada con una consecuencia positiva (CPT).

Nuestros hallazgos sobre la importancia de la rumiación son congruentes con la evidencia que muestra que la rumiación negativa media la aparición de patrones maladaptativos de recuerdos autobiográficos (Romero, Vázquez, & Sánchez, 2013) en particular, recuerdos traumáticos (Brewin, 1989). De hecho, el modelo de Brewin sobre la representación dual de las memorias traumáticas sugiere que la intrusión, un componente de la SPT, estaría relacionada con todos los contenidos almacenados en la memoria situacionalmente accesible que aún no han sido procesados ni integrados en las estructuras de los recuerdos previos. Por lo tanto, la RD estaría relacionada con el rol

adaptativo que el autor propone para la integración de estos recuerdos a la memoria verbalmente accesible (Brewin, 1989; Brewin, Dalgleish & Joseph, 1996; Brewin, 2001). La mayoría de los estudios (con la excepción del realizado por Triplett et al., 2012) han encontrado que aunque la RD no está asociada con los SPT, sí estaría relacionada con los resultados adaptativos (Benetato, 2011; García, Jaramillo et al., 2014; Morris & Shakespeare-Finch, 2011; Taku et al., 2008; Stockton et al., 2011; Taku et al., 2009; Triplett et al., 2012).

Una limitación de este estudio fue que se realizó dos años después del terremoto; por lo tanto, los resultados informados aquí podrían ser diferentes a aquellos que se pudiesen haber obtenido inmediatamente después del evento. Sin embargo, se minimizaron los sesgos de memoria ya que se les pidió a los participantes informar sobre los síntomas actuales más que los retrospectivos del malestar. Estudios anteriores han confiado en intervalos de tiempo retrospectivos similares o incluso más largos para evaluar el impacto de los eventos traumáticos (Norris, et al., 2002). Una segunda limitante fue que, aunque las relaciones causales y temporales fueron hipotetizadas a través del SEM, los datos recolectados fueron transversales y correlacionales. Por lo tanto, se debe confirmar el modelo final obtenido, que sugiere relaciones causales entre las variables, utilizando metodologías longitudinales y experimentales en los estudios a futuro.

Junto con superar estas limitantes, sería de interés que la investigación futura incorpore elementos relacionados con el proceso de regulación emocional más que con los aspectos relacionados únicamente al evento traumático. Por ejemplo, la evidencia reciente ha demostrado que la sobreproducción emocional (es decir, la coexistencia de muchas emociones negativas diferentes) es un antecedente crítico de la rumiación (Hervás & Vázquez, 2011). También se deberían identificar los factores que influyen en

la transformación de la rumiación negativa en RD y el rol de esta última en los procesos adaptativos o en el crecimiento post-traumático.

Agradecimientos

Este trabajo fue financiado en parte con una beca de pre-doctoral de la Universidad Pedro de Valdivia, una beca de investigación de la Universidad Santo Tomás y una beca CONICYT Beca Chile 5174/2013 para el primer autor. Queremos agradecer a Jessica Carney por su ayuda en la edición del manuscrito.

Referencias

Anderson, J., & Gerbing, D. (1988). Structural equation modeling in practice: A review and recommended two-step approach. *Psychological Bulletin, 103,* 411–423.

Arnoso, M., Bilbao, M A., Páez, D., Iraurgi, I., Kanyangara, P., Rimé, B., Pérez-Sales. P., & Martín-Beristain, C. (2011). Violencia colectiva y creencias básicas sobre el mundo, los otros y el yo. Impacto y reconstrucción [Collective violence and basic beliefs about the world, others and I. Impact and reconstruction]. In D. Paez, C. Martin Beristain, J. L. Gonzalez& J. De Rivera (Eds.), *Superando la violencia colectiva y construyendo cultura de paz* (pp. 247-277). Madrid: Fundamentos.

Benetato, B. (2011). Posttraumatic growth among operation enduring freedom and operation Iraqi freedom amputees. *Journal of Nursing Scholarship, 43*, 412-420.

Boals, A. & Schuettler, D. (2009). PTSD symptoms in response to traumatic and non-traumatic events: The role of respondent perception and A2 criterion. *Journal of Anxiety Disorders, 23,*458–462.

Bonanno, G. A., Westphal, M., & Mancini, A. D. (2011). Resilience to loss and potential trauma. *Annual Review of Clinical Psychology, 7*, 511-535.

Boyraz, G., & Efstathiou, N. (2011).Self-focused attention, meaning, and posttraumatic growth: The mediating role of positive and negative affect for bereaved women. *Journal of Loss and Trauma: International Perspectives on Stress & Coping, 16,* 13-32.

Brewin, C. (1989). Cognitive change processes in psychotherapy. *Psychological review, 96*(3), 379-394.

Brewin, C. (2001). A cognitive neuroscience account of posttraumatic stress disorder and its treatment. *Behaviour Research and Therapy, 39*, 373-393.

Brewin, C., Dalgleish, T., & Joseph, S. (1996). A dual representation theory of posttraumatic stress disorder. *Psychological Review, 103,* 670-686.

Burwell, R. A., & Shirk, S. R. (2007). Subtypes of rumination in adolescence: Associations between brooding, reflection, depressive symptoms, and coping. *Journal of Clinical Child and Adolescent Psychology, 36,* 56-65.

Caamaño, L., Fuentes, D., González, L., Melipillán, R., Sepúlveda, M., & Valenzuela E. (2011). Adaptación y validación de la versión chilena de la escala de impacto de evento-revisada (EIE-R) [Assessment of the impact of event scale revised for post traumatic stress disorder in Chilean subjects]. *Revista Médica de Chile, 139,* 1163-1168.

Calhoun, L. G., Cann, A., Tedeschi, R. G. & McMillan, J. (2000). A correlational test of the relationship between posttraumatic growth, religion, and cognitive processing. *Journal of Traumatic Stress, 13,* 521-527.

Calhoun, L.G., & Tedeschi, R.G. (1998). Beyond recovery from trauma: Implications for clinical practice and research. *Journal of Social Issues, 54,* 357-371.

Cann, A., Calhoun, L. G., Tedeschi, R. G., Triplett, K., Vishnevsky, T. & Lindstrom, C. (2011). Assessing posttraumatic cognitive processes: the Event Related Rumination Inventory. *Anxiety, Stress & Coping, 24,* 137-156.

Chin, W. (2010). How to write up and report PLS analysis. In V. Vinzi, W. Chin, J. Henseler, & H. Wang (Eds.), *Handbook of Partial Least Squares: Concepts, methods and applications* (pp. 655-690). Berlin, Germany: Springer-Verlag.

Cova, F., Rincón, P. & Melipillán, R. (2007). Rumiación y presencia de sintomatología ansiosa y depresiva en adolescentes [Rumination, anxiety and symptoms of depression in adolescents]. *Revista Mexicana de Psicología, 24,* 175−183.

Cova, F., Rincón, P. & Melipillán, R. (2009). Reflexión, rumiación negativa y desarrollo de sintomatología depresiva en adolescentes de sexo femenino [Reflection, negative rumination and development of depressive symptomatology in female adolescents]. *Terapia Psicológica, 27*, 155-160.

Dewaraja, R., & Kawamura, N. (2006).Trauma intensity and posttraumatic stress: Implications of the tsunami experience in Sri Lanka for the management of future disasters. *International Congress Series*, 1287, 69-73.

Echiverri, A. M., Jaeger, J. J., Chen, J. A., Moore, S. A., & Zoellner, L. A. (2011). “Dwelling in the past”: The role of rumination in the treatment of posttraumatic stress disorder. *Cognitive and Behavioral Practice, 18*, 338–349.

Ehlers, A., Mayou, R. A., & Bryant, B. (1998).Psychological predictors of chronic posttraumatic stress disorder after motor vehicle accidents. *Journal of Abnormal Psychology*, *107*, 508–519.

Ehlers, A., Mayou, R. A., & Bryant, B. (2003). Cognitive predictors of posttraumatic stress disorder in children: results of a prospective longitudinal study. *Behavior Research and Therapy*, *41*, 1–10.

Fornell, C., & Larcker, D. (1981).Evaluating structural equation models with unobservable variables and measurement error. *Journal of Marketing Research, 18*, 39-50.

García, F. (2011). Prevention of psychopathological consequences in survivor of tsunamis. In M. Mokhtari (Ed), *Tsunami: A Growing Disaster* (pp. 211-232). Rijeka, Croatia: InTech.

García, F., Cova, F. & Melipillán, R. (2013). Propiedades psicométricas del Inventario de Crecimiento Postraumático en población chilena afectada por un desastre natural. [Psychometrical properties of the Posttraumatic Growth Inventory in

Chilean population affected by a natural disaster] *Revista Mexicana de Psicología, 30* (2), 145-153.

García, F., Jaramillo, C., Martínez, A.M., Valenzuela, I., & Cova, F. (2014). Respuestas psicológicas ante un desastre natural: Estrés y crecimiento postraumático [Psychological responses to a natural disaster: posttraumatic stress and growth]. *Liberabit*.

García, F., Reyes, A., & Cova, F. (2014). Severidad del trauma, optimismo, crecimiento postraumático y bienestar en sobrevivientes de un desastre natural. [Severity of trauma, optimism, posttraumatic growth and welfare in survivors of a natural disaster] *Universitas Psychologica*, *13*(2), 15-24.

Hervás, G. y Vázquez, C. (2011). What else do you feel when you feel sad? Emotional overproduction, neuroticism and rumination. *Emotion, 11,* 881-895.

Hu, L. & Bentler, P. (1999). Cutoff criteria for fit indexes in covariance structure analysis: Conventional criteria versus new alternatives. *Structural Equation Modeling, 6*, 1-55.

Irmansyah, I., Dharmono, S., Maramis, A., & Minas, H. (2010). Determinants of psychological morbidity in survivors of the earthquake and tsunami in Aceh and Nias. *International Journal of Mental Health Systems, 4,* 4-8.

Janoff–Bulman, R. (1992). *Shattered assumptions: Towards a new psychology of trauma.* New York: The Free Press.

Janoff-Bulman, R. (2006). Schema-change perspectives on posttraumatic growth. In L.G. Calhoun & R.G. Tedeschi (Eds.), *Handbook of posttraumatic growth: Research and practice* (pp. 81–99). Mahwah, NJ: Erlbaum.

Kashdan, T., Young, K., & McKnight, P. (2012). When is rumination an adaptive mood repair strategy? Day-to-day rhythms of life in combat veterans with and without posttraumatic stress disorder. *Journal of Anxiety Disorders, 26*, 762– 768.

Leiva, M. (2011). Relevancia y prevalencia del estrés post-traumático post-terremoto como problema de salud pública en Constitución, Chile [The relevance and prevalence of post-traumatic stress after an earthquake: public health problem in Constitución, Chile]. *Revista de Salud Pública, 13*, 551-559.

Lèvy, J. P., Martín. M. T., & Román, M. V. (2006). Optimización según estructuras de covarianzas [Optimization according to covariance structures]. In J. P. Lèvy, & J. Varela (Ed.), *Modelización con estructuras de covarianzas en ciencias sociales* (pp. 11-27). La Coruña, España: Netbiblo.

Lommen, M., Sanders, A., Buck, N., & Arntz, A. (2009).Psychosocial predictors of chronic Post-Traumatic Stress Disorder in Sri Lankan tsunami survivors. *Behaviour Research and Therapy, 47*, 60-65.

MacKinnon, D. P., Lockwood, C. M., & Williams, J. (2004). Confidence limits for the indirect effect: Distribution of the product and resampling methods. *Multivariate Behavioral Research, 39*, 99–128.

Marsh, H., Balla, J., & McDonald, R. (1988). Goodness–of–fit indexes in confirmatory factor analysis: The effect of sample size. *Psychological Bulletin, 103*, 391–410.

Martens, M. (2005).The use of structural equation modeling in counseling psychology research. *The Counseling Psychologist, 33*, 269–298.

Michael, T., Halligan, S. L., Clark, D. M., & Ehlers, A. (2007). Rumination in posttraumatic stress disorder. *Depression and Anxiety, 24*, 307–317.

Ministerio Público, Chile (2011, 31 de enero). *Listado oficial de víctimas del tsunami [Official List of tsunami victims*]. Ministerio Público: Santiago de Chile.

Morris, B. & Shakespeare-Finch, J. (2011) Rumination, post-traumatic growth, and distress: Structural equation modelling with cancer survivors. *Psycho-Oncology, 20,* 1176–1183.

Morris, B., Shakespeare-Finch, J., Rieck, M., & Newberry, J. (2005). Multidimensional nature of posttraumatic growth in an Australian population. *Journal of Traumatic Stress, 18,* 575-585.

Moussa, M. (2010). *An investigation of Lebanese students' reactions, cognitive processing, coping strategies, and potential posttraumatic growth following the 2006 war in Lebanon*. (Doctoral dissertation). University of Technology, Victoria, Australia. Retrieved from http://researchbank.swinburne.edu.au/vital/access/manager/Repository/swin:16324

Muthén, L.K. & Muthén, B.O. (2010). *Mplus User's Guide* (sixth edition). Los Angeles, CA: Muthén & Muthén Copyright.

Nolen-Hoeksema, S., & Morrow, J. (1991). A prospective study of depression and post-traumatic stress symptoms after a natural disaster: The 1989 Loma Prieta earthquake. *Journal of Personality and Social Psychology, 61,* 115-121.

Norris, F., Friedman, M., Watson, P., Byrne, C., Diaz, E., & Kaniasty, K. (2002). 60,000 Disaster victims speak: Part I, an empirical review of the empirical literature, 1981–2001. *Psychiatry, 65,* 207–239.

Páez, D, Vázquez, C., Bosco, S., Gasparre, A., Iraurgi, I. & Sezibera, V. (2011). Crecimiento post estrés y post trauma: Posibles aspectos positivos y beneficiosos de la respuesta a los hechos traumáticos [Post-trauma and post-stress growth: Possible positive and beneficial aspects of the response to traumatic events]. In: D. Páez, C. Martín Beristain, J. L. González, N. Basabe & J. De Rivera (Eds.).

Superando la violencia colectiva y construyendo cultura de paz (pp. 311-339). Madrid: Fundamentos

Papageorgiou, C., & Wells, A. (2003). An empirical test of a clinical metacognitive model of rumination and depression. *Cognitive Therapy and Research, 27,* 261-273.

Park, C. (2010). Making sense of the meaning literature: an integrative review of meaning making and its effects on adjustment to stressful life events. *Psychological Bulletin*, *136*, 257–301.

Pimentel, M. & Cova, F. (2011). Efectos de la rumiación y la preocupación en el desarrollo de sintomatología depresiva y ansiosa en estudiantes universitarios de la ciudad de Concepción, Chile [Depressive rumination and worry in the development of depressive and anxious symptoms in university students]. *Terapia Psicológica, 29,* 43-52.

Raes, F., & Hermans, D. (2008). On the mediating role of subtypes of rumination in the relationship between childhood emotional abuse and depressed mood: Brooding vs. reflection. *Depression and Anxiety*, *25*, 1067–1070.

Romero, N., Vázquez, C., & Sánchez, A. (2013). Rumination and specificity of autobiographical memory in dysphoria. *Memory*. doi:10.1080/09658211.2013.811254.

Roussos, A., Goenjian, A., Steinberg, A., Sotiropoulou, C., Kakaki, M., Kabakos, C., Karagianni, S., & Manouras, V. (2005). Posttraumatic stress and depressive reactions among children and adolescents after the 1999 earthquake in Ano Liosia, Greece. *American Journal of Psychiatry, 162,* 530-537.

Russell, D. W., Kahn, J. H., Spoth, R., & Altmaier, E. M. (1998). Analyzing data from experimental studies: A latent variable structural equation modeling approach. *Journal of Counseling Psychology, 45*, 18–29.

Shakespeare-Finch, J., & Lurie-Beck, J. (2014). A meta-analytic clarification of the relationship between posttraumatic growth and symptoms of posttraumatic distress disorder. *Journal of Anxiety Disorders*, *28*(2), 223-229.

Stockton, H., Hunt, N. & Joseph, S. (2011). Cognitive processing, rumination, and posttraumatic growth. *Journal of Traumatic Stress, 24*, 85–92.

Sumer, N., Karanci, N., Berument, S., & Gunes, H. (2005). Personal resources, coping self-efficacy, and quake exposure as predictors of psychological distress following the 1999 earthquake in Turkey. *Journal of Traumatic Stress*, *18,* 331–342.

Taku, K., Calhoun, L., Cann, A., & Tedeschi, R. (2008). The role of rumination in the coexistence of distress and posttraumatic growth among bereaved Japanese university students. *Death Studies, 32*, 428-444.

Taku, K., Cann, A., Tedeschi, R. & Calhoun, L. (2009). Intrusive versus deliberate rumination in posttraumatic growth across U.S. and Japanese samples. *Anxiety, Stress, & Coping, 22*, 129-136.

Tedeschi, R.G. & Calhoun, L.G. (1996). The Posttraumatic Growth Inventory: Measuring the positive legacy of trauma. *Journal of Traumatic Stress, 9,* 455–471.

Tedeschi, R. G. & Calhoun, L.G. (2004). Posttraumatic growth: Conceptual foundations and empirical evidence. *Psychological Inquiry, 15,* 1-18.

Tedeschi, R. G., & Kilmer, R. (2005). Assessing strengths, resilience, and growth to guide clinical interventions. *Professional Psychology: Research and Practice, 36,* 230-237.

Treynor, W., Gonzalez, R. & Nolen-Hoeksema, S. (2003). Rumination reconsidered: A psychometric analysis. *Cognitive Therapy & Research*, *27*, 247–259.

Triplett, K., Tedeschi, R. G., Cann, A., Calhoun, L. G., & Reeve, C. (2012).Posttraumatic growth, meaning in life, and life satisfaction in response to trauma. *Psychological Trauma: Theory, Research, Practice, and Policy, 4*, 400-410.

Tural, U., Aybar Tolun, H., Karakaya, I., Erol, Y., Yildiz, M., & Erdoğan, S. (2001). Predictors of current comorbid psychiatric disorders with posttraumatic stress disorder in earthquake survivors. *Turkish Journal of Psychiatry, 12*, 175-183.

United States Geological Survey (2010). *Historic World Earthquakes.* Retrieved on January 31, 2013 at: http://earthquake.usgs.gov/earthquakes/world/historical_mag.php.

Ullman, J. B. (2007). Structural equation modeling. In B. G. Tabachnick & L. S. Fidell (Eds.), *Using multivariate statistics,* (5th ed., pp. 676–780). Boston, MA: Allyn & Bacon.

Vázquez, C. (2013). A new look at trauma: From vulnerability models to resilience and positive changes. In K.A. Moore, K. Kaniasty, P. Buchwald, & A. Sese (Eds), *Stress and Anxiety – Applications to health and well-being, work stressors and assessment* (pp. 27-40). Logos Verlag: Berlin.

Vázquez, C., Hervás, G., & Pérez-Sales, P. (2008). Chronic thought suppression as a vulnerability factor to posttraumatic symptoms: data from the Madrid March 11, 2004 terrorist attack. *Journal of Anxiety Disorders, 22*, 1226-1236

Wahlström, L., Michélsen, H., Schulman, A., & Backheden, M. (2008). Different types of exposure to the 2004 tsunami are associated with different levels of psychological distress and posttraumatic stress. *Journal of Traumatic Stress, 21*, 463–470.

Walter, M. & Bates, G (2012). Posttraumatic growth and recovery from post traumatic stress disorder. In V. Olisah (Ed.), *Essential notes in Psychiatry* (pp. 143-186). Rijeka, Croatia: InTech.

Weiss, T. & Berger, R. (2010). *Posttraumatic growth and culturally competent practice: Lessons learned from around the globe*. New York: Wiley.

Weiss, D., & Marmar, C. (1997). The Impact of Event Scale-Revised. In J. P. Wilson, & T. M. Keane (Eds.), *Assessing psychological trauma and PTSD: A practitioner's handbook* (pp. 399-411). New York: Guilford Press.

Werts, C., Linn, R., & Joreskog, K. (1974). Interclass reliability estimates: Testing structural assumptions. *Education and Psychological Measurement, 34,* 25-33.

Xu, J., & Song, X. (2011). Posttraumatic stress disorder among survivors of the Wenchuan earthquake 1 year after: Prevalence and risk factors. *Comprehensive Psychiatry, 52*, 431-437.

Yu, C. Y. (2002). *Evaluating cutoff criteria of model fit Indices for latent variable models with binary and continuous outcomes*. (Unpublished Doctoral Dissertation). University of California, Los Angeles, USA. Retrieved from http://www.statmodel.com/download/Yudissertation.pdf

Yuan, K.-H., & Bentler, P. M. (2007). Structural equation modeling. In C. R. Rao & S. Sinharay (Eds.), *Handbook of statistics 26: Psychometrics* (pp. 297–358). Amsterdam: North-Holland.

Tabla 1:

Medias, Desviaciones Estándares, Confiabilidad Compuesta (CC), AVE, √AVE y correlaciones entre las Medidas Principales del Estudio

	Medidas	Mín.	Máx.	Media	DE	CC	AVE	√AVE	2	3	4	5
1	SS	0	8	5,58	1,99	0,90	0,82	0,91	0,49***	0,43***	0,50***	0,32***
2	R. Neg.	0	21	8,95	5,73	0,90	0,57	0,76	-	0,54***	0,73***	0,28***
3	RD	0	12	6,21	3,19	0,88	0,64	0,80		-	0,55	0,44***
4	SPT	0	36	10,76	9,60	0,93	0,68	0,83			-	0,25***
5	CPT	0	45	29,22	11,19	0,93	0,61	0,78				-

SS=Severidad Subjetiva; RD=Rumiación Deliberada; SPT=Síntomas Post-Traumáticos; CPT= Crecimiento Post-Traumático.
*** p< 0,001.

Tabla 2:

Cargas Factoriales Estandarizadas para variables latentes en el modelo de medición.

Variable latente	Indicadores factoriales	S.E.	*Z*	Cargas factoriales
Severidad Subjetiva	S1	-	-	0,71
	S2	0,160	9,00	0,90
Rumiación negativa	BPAR1	-	-	0,86
	BPAR2	0,031	21,27	0,84
	BPAR3	0,033	19,61	0,88
Rumiación Deliberada	RUM1	-	-	0,73
	RUM2	0,088	12,46	0,75
	RUM3	0,092	13,03	0,79
	RUM4	0,089	10,64	0,63
Síntomas Post-traumáticos	Evitación	-	-	0,89
	Hiperactivación	0,044	23,16	0,87
	Intrusión	0,038	26,13	0,93
Crecimiento Post-traumático	Auto-Percepción	-	-	0,87
	Relaciones Interpersonales	0,081	16,99	0,89
	Filosofía de Vida	0,048	12,99	0,65

Nota: Todos los estimados de los parámetros son significativos a $p<0{,}001$

Tabla 3:

Índices de ajuste para el Modelo 1 y el Modelo 2

Modelo	χ^2 *(gl)*	*p*	χ^2/gl	*CFI*	*TLI*	*PNFI*	*RMSEA (IC 90%)*
Modelo 1	169,97 (85)	0,00	2,00	0,97	0,97	0,77	0,053 (0,04-0,07)
Modelo 2	146,96 (83)	0,00	1,77	0,98	0,97	0,75	0,047 (0,03-0,06)

Tabla 4:
Los efectos indirectos estandarizados, el IC de 95% para los estimados (límite inferior y superior), sus errores estándares y los valores p

Variable	Efectos indirectos (IC 95%)	EE	p
SS→CPT	0,559 / 1,400	0,931	0,001
R. NEG. →CPT	0,185 / 0,432	0,291	0,001
SS→SPT	1,211 / 2,039	1,59	0,001

SS=Severidad Subjetiva; CPT=Crecimiento Post-traumático; SPT=Síntomas Post-traumáticos

Figura 1: Modelo 1 hipotetizado

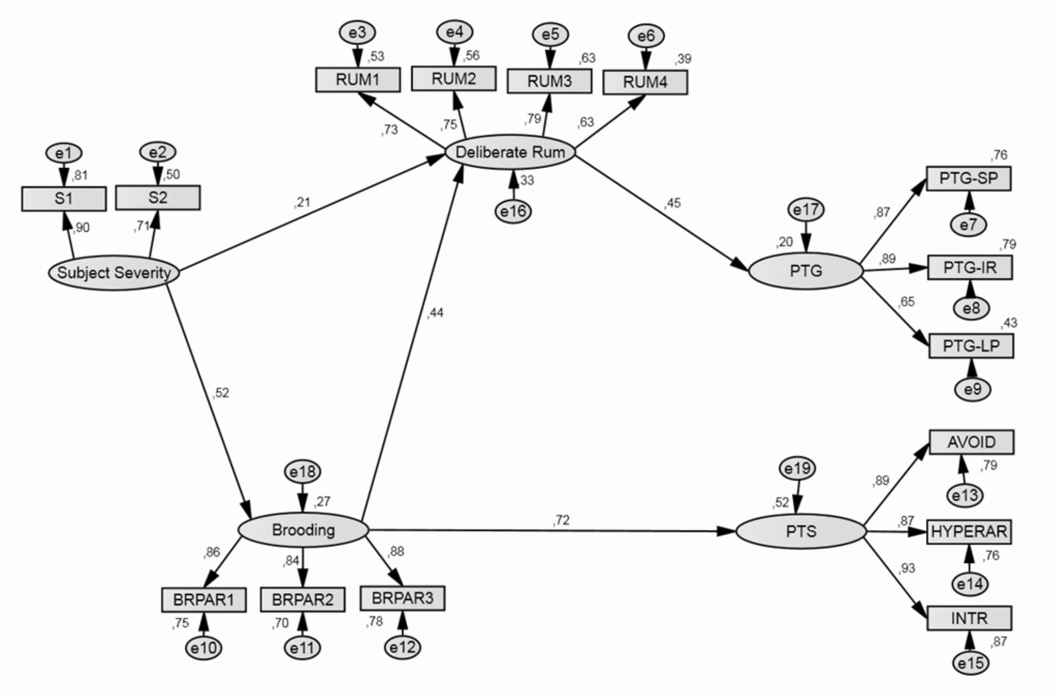

Figura 2: Modelo 2 alternativo

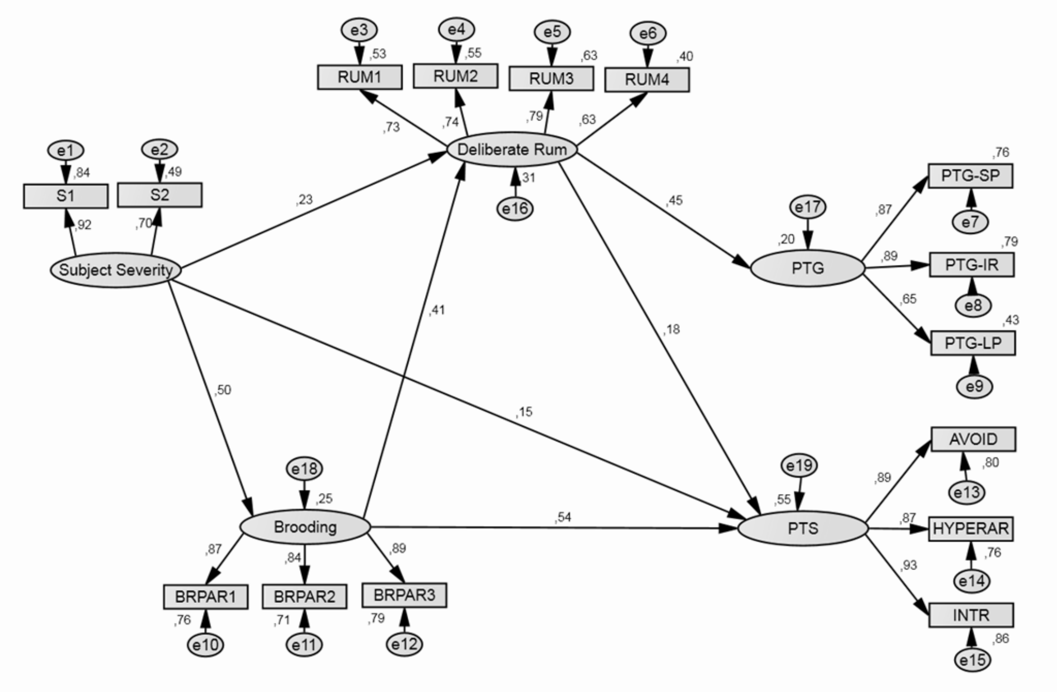

Supplement: Trauma or growth after a natural disaster? The mediating role of rumination processes [file EJPT-6-26557-s003.pdf]
