# Supplementary material for: Trauma or growth after a natural disaster? The mediating role of rumination processes
Source: Eur J Psychotraumatol. 2015 Jul 31;6:10.3402/ejpt.v6.26557. doi: 10.3402/ejpt.v6.26557 (PMC4522433; doi:10.3402/ejpt.v6.26557)
Supplement: Trauma or growth after a natural disaster? The mediating role of rumination processes [file EJPT-6-26557-s004.pdf]

Trauma oder Wachstum nach einer Naturkatastrophe? Die mediierende Rolle des Grübelprozesses

Felipe E. García, Félix Cova, Paulina Rincón, Carmelo Vázquez

Zusammenfassung:

Das Ziel der Studie war ein kognitives Model von posttraumatischen Symptomen (PTS) und posttraumatischen Wachstums (PTW), nach dem Erleben einer Naturkatastrophe, zu überprüfen. Es wurde vermutet, dass der subjektive Schweregrad des Traumas mit dem Schweregrad der PTS im Zusammenhang steht, dieser Zusammenhang jedoch von Sinnieren und kognitive Strategien, in Verbindung mit wiederholten negativen Inhalten in den Gedanken, vollständig mediiert wird. Auch der Zusammenhang zwischen dem subjektiven Schweregrad des Traumas und PTW wird durch Grübeln und kognitive Strategien, fokussiert auf den bewussten Umgang mit der Katastrophe, vollständig mediiert. Um das kognitive Model zu überprüfen wurden Erwachsene (N=351), welche durch das Erdbeben und Tsunami am 27. Februar 2010 in Chile ihr Haus verloren haben, selektiert. Ein Strukturgleichungsmodel wurde erstellt um die Daten zu analysieren. Das errechnete Model hatte eine angemessene Passung und zeigte, dass Sinnieren den Zusammenhang zwischen dem subjektiven Schweregrad des Traumas und posttraumatische Symptome vollständig mediiert und Grübeln den Zusammenhang zwischen dem subjektiven Schweregrad des Traumas, Sinnieren und PTW mediiert. Das Ergebnis unterstreicht die wichtige Rolle des Grübelprozesses und Inhalts in Zusammenhang mit Trauma, PTS und PTW. Die Bedeutung der Resultate für ein vollständigeres Model über den Schweregrad des Traumas wird diskutiert.

Schlagwörter: benefit finding, Distress, Erdbeben, Naturkatastrophe, Grübeln, posttraumatische Stresssymptome, Tsunami

Name of translator: FP

Def.: brooding is the specific component of rumination that favors more negative emotional consequences

Mögliche Übersetzungen: grüblerisch, grübelnd, brütend, sinnierend, quälerisch grübelnd

Citation: European Journal of Psychotraumatology 2015, **6**: 26557 - http://dx.doi.org/10.3402/ejpt.v6.26557
