# Supplementary material for: Trauma or growth after a natural disaster? The mediating role of rumination processes
Source: Eur J Psychotraumatol. 2015 Jul 31;6:10.3402/ejpt.v6.26557. doi: 10.3402/ejpt.v6.26557 (PMC4522433; doi:10.3402/ejpt.v6.26557)
Supplement: Trauma or growth after a natural disaster? The mediating role of rumination processes [file EJPT-6-26557-s005.pdf]

Title 在自然灾难后发生创伤还是成长？思维反刍过程的中介作用

Authors: Felipe E. García, Félix Cova, Paulina Rincón, Carmelo Vázquez

本研究的目标是评估一个暴露于自然灾难后的创伤后症状（PTS）和创伤后成长（PTG）相关的认知模型。假设认为尽管对灾难的主观严重程度估计与 PTS 的严重程度相关，但是这种相关涉及以下中介因素：沉思及认知策略，该认知策略牵涉头脑中不断重复负性思维内容。而且，严重程度估计和 PTG 完全受蓄意思维反刍中介，该认知策略涉及意识层面上主动聚焦于灾难事件。为了评价认知模型，我们选择了成年人（N=351），他们在智利 2010 年 2 月 27 日的地震和海啸中流离失所。使用结构方程模式来分析数据。结果是该模型有恰当的拟合优度指数，证实沉思完全介导了主观严重程度和 PTS，蓄意思维反刍完全介导了主观严重程度、沉思和 PTG。这些结果提示思维反刍的内容和过程在创伤的主观严重程度、PTS 和 PTG 之间起中介作用。我们也讨论了这些结果在形成一个创伤后症状综合模型方面的意义。

Keywords: 利益查找、不幸、地震、自然灾害、思维反刍、创伤后应激症状、海啸

Name of translator: Xuan JU, Zhonglin Tan

Citation: European Journal of Psychotraumatology 2015, **6**: 26557 - http://dx.doi.org/10.3402/ejpt.v6.26557
